# Incidence of Tuberculosis and Amyloidosis among Sudanese Patients Presented with Enlarged Nodes

**DOI:** 10.1155/2014/832029

**Published:** 2014-03-30

**Authors:** Ahmed Abdalla Agab Eldour, Entisar Najeeb Mohmed Salih, Hussain Gadelkarim Ahmed

**Affiliations:** ^1^Department of Pathology, Faculty of Medicine, University of Kordofan, El-Obied, Sudan; ^2^Department of Pathology, College of Medicine, University of Hail, Hail 2440, Saudi Arabia

## Abstract

*Objectives*. To determine the incidence of tuberculous lymphadenitis and amyloidosis in enlarged lymph nodes (LN). *Methodology*. A retrospective study was carried out in the department of pathology at the Faculty of Medicine, University of Kordofan, during one-year period. The study included a group of 103 patients with lymphadenopathy. *Results*. Of the 103 cases with enlarged neck nodes, 35 (34%) had tuberculous lymphadenitis. Sixteen (46%) cases were males and 19 (54%) cases were females. Out of the 103 patients, amyloidosis was diagnosed in 9/103 (8.7%). Out of the 9 positive cases, 2/9 (22.2%) were males and 7/9 (77.8%) were females. *Conclusion*. There is high incidence of tuberculous lymphadenitis in patients with enlarged LN in developing countries like North Sudan. Amyloidosis should not be ignored when investigating enlarged LN.

## 1. Introduction

Tuberculous lymphadenitis is the most frequent presentations of extra pulmonary tuberculosis (TB) [1]. TB is responsible for up to 43% of peripheral lymphadenopathy in the developing world [2]. The epidemiology and diagnostic aspects of tuberculous lymphadenitis differ according to geographic region and the burden of TB and HIV infection [3]. Globally the incidence of all forms of TB is decreasing. However, the rate is not similar across all WHO regions. In the Africa region it is decreasing slowly by 1.8% per year [4].

Tuberculous lymphadenitis (or tuberculous adenitis) is a chronic specific granulomatous inflammation of the lymph node with caseation necrosis, caused by infection with* Mycobacterium tuberculosis* or* Mycobacterium bovis* [5, 6].

Sudan is a large country with a diverse population and history of civil conflict. Poverty levels are high with a gross national income per capita of less than two thousand dollars. The country has a high burden of tuberculosis (TB) with an estimated 50,000 incident cases during 2009, when the estimated prevalence was 209 cases per 100,000 of the population [7]. The estimated adult HIV prevalence of 1.5% remains lower than that of its African neighbours to the south and a report from 2002 suggested 4% of tuberculosis patients were coinfected with HIV [8]. However, there is a lack of studies screened tuberculous lymphadenitis reported from Sudan, in general and Kordofan state in particular; thus, one of the aims of the present study was to find out the incidence of tuberculous lymphadenitis in Kordofan State.

Amyloidosis is a disease characterized by an extracellular deposition of the fibrous protein either involving multiple organ systems (systemic amyloidosis) or restricted to a single-tissue site (localized amyloidosis) [9, 10]. The disease nomenclature is based on the precursors of the amyloid fibrils for which at least 28 different proteins have been identified [11–13].

The diagnosis of amyloidosis is based on the demonstration of amyloid fibrils in a tissue biopsy. In AL amyloidosis, the presence of a monoclonal immunoglobulin can usually be demonstrated in serum or urine samples. The symptoms of amyloidosis arise in the critical organs where amyloid accumulates, including the heart, kidney, liver, and peripheral nerves [12]. Staining of tissue with Congo red shows the characteristic red staining of amyloid in normal light but apple green birefringence under polarized light microscopy, which remains the gold standard for diagnosis [14]. Later electron microscopy demonstrated that amyloid was a fibrous protein [15] with a unique cross *β* pattern on X-ray diffraction [16, 17]. The amyloidoses are thus a heterogeneous group of disorders related by the deposition of proteins that share a remarkably similar and stable core structure of *β* sheets. Therefore, in the present study we screened biopsies obtained from patients with lymphadenopathy for the presence of amyloid materials using Congo Red histochemical method.

## 2. Materials and Methods

This is a retrospective study to screen patients with enlarged LN for the presence of amyloid materials and* Mycobacterium tuberculosis*. The study was conducted in El-Obied, Sudan, during the period from 2010 to 2011. One hundred and three LN biopsies were retrieved from previously referred LN biopsies (during the period from 2008 to 2011), for histopathology. All specimens were formalin-fixed and paraffin wax processed tissues. Information regarding each patient was obtained from each patient's file. The specimens were fixed in 10% formalin and then processed by tissue processing machine using a schedule adopting 24-hour scheduling. Three 5-micron thickness sections were obtained from each patient's block using Rotary Microtome. Of the 3 sections, each one was stained with Haematoxylin and Eosin procedure (H&E), the second with Zeil-Neilson (ZN), and the third with Congo red. Those showing histopathological pattern containing giant cells + granuloma + caseation were considered as strong evidence for diagnosis of TB lymphadenitis.

### 2.1. Ethical Consent

The study was submitted and approved by the Department Research Board of Faculty of Medicine and Medical science, University of Kordofan, El-Obied, Sudan, and Histopathology Laboratory at El-Obied regional Laboratory.

### 2.2. Statistical Analysis

For all statistical analyses, the SPSS (version 10) was used. Person Chi-square test with 95% confidence level was used. *P* values of 0.05 or less were regarded as statistically significant.

## 3. Results

**Figure 1:**
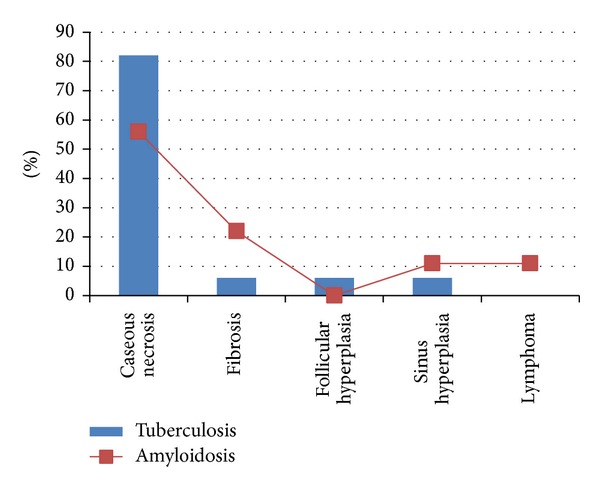
Description of tuberculosis and amyloidosis positive patients by histopathology.

**Figure 2:**
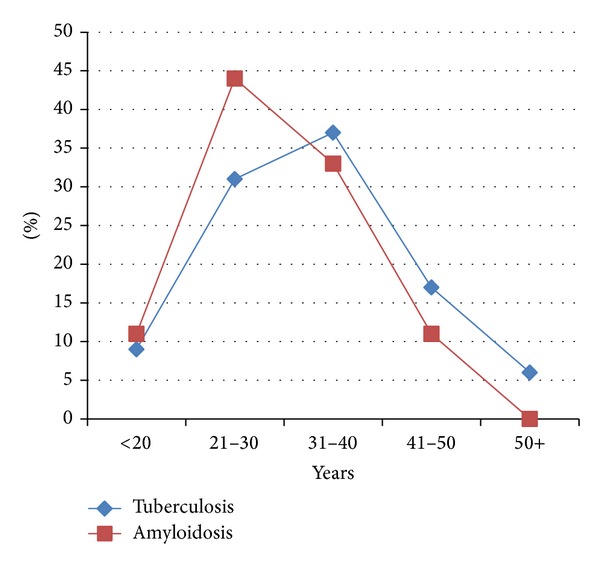
Description tuberculosis positive patients by age.

In this study 103 samples obtained from patients with enlarged LNs were retrospectively investigated for the presence of tuberculosis and amyloidosis. The male to female ratio was 1.00 : 1.102. Their ages ranged from 13 to 65 years with a mean age of 33 years old. Of the 103 patients, tuberculosis was diagnosed in 35/103 (34%). Out of the 35 positive cases, 16/35 (45.7%) were males and 19/35 (54.3%) were females. According to histopathological diagnosis, the great majority of tuberculosis cases were diagnosed as having caseous necrosis representing 29/35 (82.5%), followed by fibrosis, follicular hyperplasia, and sinus hyperplasia, constituting 2/35 (5.7%) for each, as indicated in Figure 1. In regard to age, most positive cases were identified among age range 31–40 years constituting 13/35 followed by 20–30, 41–50, <20, and 50+, representing, 11, 6, 3, and 2, respectively, as indicated in Figure 2.

Out of the 103 patients, amyloidosis was diagnosed in 9/103 (8.7%). Out of the 9 positive cases, 2/9 (22.2%) were males and 7/9 (77.8%) were females. According to histopathological diagnosis, the great majority of amyloidosis cases were diagnosed as having caseous necrosis representing 5/9 (55.5%), followed by fibrosis, sinus hyperplasia, and lymphoma, constituting 2/9 (22.2%), 1/9 (11.1%), and (11.1%), respectively, as indicated in Figure 1. Notably, the 5 positive caseous necrosis cases were also found positive for tuberculosis. In regard to age, most positive cases were identified among age range 21–30 years constituting 4/9 followed by 31–40, 41–50, <20, and 50+, representing, 3, 1, 1, and 0, respectively, as indicated in Figure 2.

## 4. Discussion

Tuberculosis (TB) is one of the most common causes of mortality from an infectious disease, and it represents alarming challenges to global health. Extrapulmonary TB is a major health problem, since it is difficult to diagnose and to monitor its treatment. The epidemiology of tuberculous lymphadenitis varies greatly in different countries. The highest proportion was reported from Cambodia (34.2%) and the lowest from China (0.69%) [18]. What is more is that the extra pulmonary TB is established in 10–34% of non-HIV cases, whereas it occurs in 50–70% of patients coinfected with HIV [19]. In this study the diagnosis of TB lymphadenitis was established when histopathological section from the biopsy showed characteristics of TB or positive ZN stain. In fact this measurement suffers from misdiagnosis with other inflammatory conditions that may give similar histopathological picture. Although Zeil-Neelsen (ZN) stain and even fluorescence have low sensitivity, their combined use may confirm the diagnosis of TB lymphadenitis when the molecular diagnosis is unaffordable or non-available.

However, most available data regarding TB is referring to pulmonary TB and there is a lack of data regarding prevalence of TB lymphadenitis from Sudan. The prevalence of TB in Sudan was 209 cases per 100,000 of the population and 50,000 incident cases during 2009 [20]. The estimated adult HIV prevalence of 1.5% remains lower than that of its African neighbors to the south and a report from 2002 suggested 4% of tuberculosis patients were coinfected with HIV [8]. In a study from Sudan to determine whether* Mycobacterium tuberculosis* infection spreads through the blood to different lymph-node groups in patients with tuberculous lymphadenitis, a close link is indicated. The presence of* M. tuberculosis* DNA correlated strongly to multiple lymph-node involvement [OR (odds ratio) = 96.7, 95% confidence interval (CI) 9.0–1,039] and to caseating-granulomatous and predominantly necrotic cytomorphological categories [OR = 70, 95% confidence interval (CI) 7.0–703]. Of the 52 enrolled patients, 30/52 (57.7%) were with FNAC diagnosis of tuberculous lymphadenitis and positive PCR for* M. tuberculosis* complex [21], it is higher than the incidence of TB lymphadenitis as reported in the present study. A recent study from Sudan examined 222 patients suspected to have tuberculous lymphadenitis. 57 patients biopsies were taken and stained by H and E. About 94.6% of the cytology shows positive result for TB. The mycobacterium grows in 88% of the culture media. Acid fast bacilli were seen in 61 patients (41.6%). Serological test was positive in 68% of the patients. Studied females were more than males (M : F = 1 : 1.2) [22], which was similar to our findings.

However, incidence of TB lymphadenitis in El-Obied is high. Implementation of TB control programs in poor and limited resources regions like El-Obied is highly recommended.

Amyloidosis is a heterogeneous disease caused by deposition of amyloid fibrils in organs progressively replacing their original cells [23]. What is worse is that no any incidence data are available and most survival data are limited to specialist clinics. There are limited data on the incidence of amyloidosis probably because of the rarity and heterogeneity of the condition. The widely global exchanged studies were those reports by Kyle and coworkers [24]; they found that the incidence for AL amyloidosis in United States was 9 per million person-years. Another larger study included amyloidosis cases for years 2001 to 2008, a total of 949 patients were identified, giving an incidence of 8.29 per million person-years [23].

However, there is a complete absence of data regarding amyloidosis from Sudan, and this might be the first report in this context. The present study helps to fill in the gap of data regarding the incidence of amyloidosis. The advantages include generally awareness in a country of limited access to medical services of low diagnostic standards. Many patients might be discharged more than once without distinct diagnosis of amyloidosis. A recent study has shown that the incidence of amyloidosis in sub-Saharan Africa ranges from 0.28 to 0.57% in autopsy series. AA amyloidosis is the most frequent, found in 42 to 66% of amyloidoses. Chronic infections, especially tuberculosis, are the main cause. AL amyloidosis is found in 21 to 34% of amyloidosis cases, half of them due to myeloma [25]. One of the major limitations in this study was the use of Congo red only in identification of amyloid materials, which does not identify the different types. The use of immunohistochemistry for the positive cases might be useful, since it can provide some information about the dominant amyloid type.

To the best of our knowledge, this is the first report about amyloidosis from Sudan. However, the relationship between amyloidosis and tuberculosis was well established [26, 27]. Amyloidosis (AA) can occur secondary to several infectious, inflammatory, and malignant conditions and is caused by the degradation of the acute-phase protein that is produced in response to inflammatory conditions [28, 29]. Common causative infections include TB, bronchiectasis, osteomyelitis, and leprosy [30, 31].

Further studies will allow a better assessment of the characteristics of amyloidosis in Sudan in general and El-Obied in particular.
